# Pre-Metabolic Syndrome and Incidence of Type 2 Diabetes and Hypertension: From the Korean Genome and Epidemiology Study

**DOI:** 10.3390/jpm11080700

**Published:** 2021-07-22

**Authors:** A-Ra Cho, Yu-Jin Kwon, Jong-Koo Kim

**Affiliations:** 1 Department of Family Medicine, Yongin Severance Hospital, Yonsei University College of Medicine, Yongin-si 16995, Korea; ARA1713@yuhs.ac; 2 Department of Family Medicine, Wonju College of Medicine, Yonsei University, Won-Ju 26426, Korea

**Keywords:** cohort study, hypertension, metabolic syndrome, pre-metabolic syndrome, type 2 diabetes mellitus

## Abstract

The aim of this study was to investigate the prevalence of premetabolic syndrome (pre-MetSyn) and its components and to longitudinally examine their association with new-onset type 2 diabetes (T2D) or hypertension. A total of 4037 men and 4400 women aged 40 to 69 years were selected from the Korean Genome and Epidemiology Study, observed from 2001 to 2014. Pre-MetSyn was defined as the presence of one or two components of MetSyn (B, elevated blood pressure; G, elevated glucose; H, low HDL-cholesterol; T, elevated triglycerides; W, increased waist circumference). The prevalence of pre-MetSyn was higher than that of non-MetSyn and MetSyn in both men and women. In multivariate Cox regression analyses, G, T, G+T, W+G, B+G, B+T, W+T, B+H, and H+T in men and G, T, G+H, B+T, and H+T in women were significantly associated with new-onset T2D. B, W, B+H, B+T, W+H, and W+T in men and B, B+T, B+H, B+W, and W+H in women were significantly associated with new-onset hypertension. The prevalence of pre-MetSyn components and their associations with new-onset T2D or hypertension differed according to sex and disease. Our results suggest that specific phenotypes of pre-MetSyn may be important factors for predicting and preventing the development of T2D and hypertension.

## 1. Introduction

Metabolic syndrome (MetSyn) was first described by Reaven in 1988 [1] under the name of “Syndrome X”, which included glucose intolerance, hyperinsulinemia, increased very-low-density lipoprotein (VLDL) and triglyceride levels (TG), decreased high-density lipoprotein (HDL) cholesterol level, and hypertension. Since then, several groups of experts have reached some agreement about the components of MetSyn [2,3,4]; however, there remains disagreement regarding how to best define this syndrome. For example, MetSyn statements from the International Diabetes Federation and the American Heart Association/National Heart, Lung, and Blood Institute differed regarding whether to consider waist circumference an obligatory component [3,4]. In 2009, expert groups agreed that three of five abnormal findings (increased waist circumference, blood pressure elevation, low HDL-cholesterol, high triglycerides, and hyperglycemia) were sufficient to diagnose MetSyn [5].

MetSyn is associated with an increased risk of type 2 diabetes (T2D), hypertension, cardiovascular disease (CVD), and all-cause mortality [6,7,8,9]. Since MetSyn is a constellation of different physiologic conditions, many researchers have extended the simple association between the overall syndrome (i.e., absence or presence of MetSyn) and chronic diseases to more heterogeneous relationships between the number of MetSyn components or specific combinations of these components and the outcomes of T2D, CVD, and all-cause mortality [7,9,10,11,12,13]. Furthermore, several studies have focused on pre-MetSyn, the precursor of MetSyn, because of the association between individual MetSyn components and an increased risk of these adverse outcomes [14,15,16]. A recent study [17] suggested that the pre-MetSyn state could be the best time to initiate effective treatment. As pre-MetSyn can progress to MetSyn or its components, it may represent the critical transition phase for the development of clinical diseases. For example, subjects with an increased waist circumference but without hyperglycemia may subsequently develop T2D [10,11]. Although most researchers have agreed that pre-MetSyn represents a stage at which the existing MetSyn criteria are not met, detailed criteria for defining pre-MetSyn (such as the number of MetSyn components) have not yet converged. Some groups defined pre-MetSyn as the presence of two MetSyn components [14,16], whereas another group defined pre-MetSyn as the presence of one or two components [15]. Furthermore, although a few studies have found associations between individual MetSyn components and T2D or CVD, the findings have been inconsistent, and there is a need for studies focusing on combinations of MetSyn components in patients with pre-MetSyn [7,10,11].

Understanding the prevalence of each MetSyn component and their associations with chronic diseases provides a powerful tool to individualize treatment regimens and identify individuals with an increased risk of complications at diagnosis. Therefore, we investigated the prevalence patterns of MetSyn components in a large population-based cohort of community-dwellers from Korea. Furthermore, we examined whether different combinations of MetSyn components in patients with pre-MetSyn are differentially associated with new-onset T2D or hypertension in this cohort when observed over a 12-year period.

## 2. Materials and Methods

### 2.1. Study Population

The study population consisted of individuals included in the Korean Genome and Epidemiology Study Ansan and Ansung (KoGES Ansan and Ansung or KoGES). KoGES was a large population-based cohort study consisting of male and female community-dwellers, aged 40–69 years at baseline. This study was conducted biennially from 2001–2002 (baseline) to 2013–2014 (sixth follow-up). A total of 10,030 adults aged 40–69 years were included in KoGES. We excluded participants with missing data for medical history (*n* = 4); exercise, smoking, and alcohol history (*n* = 609); anthropometric values (*n* = 21); and laboratory tests (*n* = 284). We also excluded participants lost to follow-up after the baseline evaluations (*n* = 913). A total of 8437 (4037 men and 4400 women) adults were thus included in the final study (Supplementary Figure S1). All participants provided written informed consent before participating in any study-related procedures. This cohort study was conducted in accordance with the principles of the Declaration of Helsinki as revised in 2008. The protocol for this study was approved by the Institutional Review Board (IRB) of Yongin Severance Hospital (IRB number: 9-2020-0043).

### 2.2. Data Collection

Body mass index (BMI) was calculated as the body weight divided by height squared (kg/m^2^). Waist circumference was measured with the patient in the standing position using a measuring tape placed midway between the inferior margin of the last rib and the iliac crest. After fasting for at least 8 h, fasting glucose, triglycerides, and HDL-cholesterol levels were analyzed according to the KoGES protocol. Blood pressure was measured at least twice in the sitting position using a mercury sphygmomanometer. Self-reported questionnaires were used to obtain information about smoking status, alcohol intake, medications, and the presence of hypertension or T2D. Smoking was defined as being a current smoker and having smoked at least 100 cigarettes over his/her lifetime. The amount of alcohol intake per day was recorded. Because information about regular exercise was not available in the baseline survey during KoGES, we could not consider the effects of regular exercise in the current study [18].

### 2.3. Definition of MetSyn, New-Onset T2D, and New-Onset Hypertension

MetSyn components were defined according to the revised version of the National Cholesterol Education Program Adult Treatment Panel III criteria [4]. The component definitions, with their corresponding 1-letter abbreviations, were as follows: (1) waist circumference ≥90 cm in men and ≥80 cm in women (in accordance with the International Obesity Task Force criteria for the Asian-Pacific population) (W); (2) serum triglycerides ≥ 150 mg/dL or drug treatment for elevated triglycerides (T); (3) serum HDL-cholesterol < 40 mg/dL in men and <50 mg/dL in women or drug treatment for reduced HDL-cholesterol (H); (4) systolic blood pressure (SBP) ≥ 130 mm Hg, diastolic blood pressure (DBP) ≥ 85 mm Hg, or receiving antihypertensive medications (B); and (5) fasting blood glucose ≥ 100 mg/dL or receiving antidiabetic medications (G). We set a total of 15 groups of MetSyn components representing pre-MetSyn: 5 groups for each of the 5 components (W, T, H, B, and G) and 10 combination groups (5C2) consisting of two MetSyn components (B+W, B+G, B+H, B+T, W+G, W+H, W+T, G+H, G+T, and H+T).

We defined MetSyn as having three or more MetSyn components. T2D was defined as a fasting blood glucose ≥ 125 mg/dL, hemoglobin A1C ≥ 6.5%, or taking antidiabetic medications. Hypertension was defined as an SBP ≥ 140 mm Hg, DBP ≥ 90 mmHg, or receiving antihypertensive medications. The endpoints of this study were new-onset T2D and new-onset hypertension.

### 2.4. Statistical Analyses

Data are presented as mean ± standard deviation (SD) or percentage (%). For continuous variables, the independent *t*-test was used to compare differences between groups. For categorical variables, the chi-squared test was used to compare differences between groups. Gender-specific analyses were conducted for all statistical analyses. Hazard ratios (HRs) and 95% confidence intervals (CIs) were derivated using multivariate Cox regression analysis, after adjusting for age, BMI, smoking, and alcohol intake. We performed post-hoc analyses for the multivariate Cox regression analysis. Specifically, we selected subjects with two of the 15 single MetSyn components or two-component combinations and subsequently conducted multivariate Cox regression with these subjects. We iterated this for all possible unique pairs (15C2 = 105) and reported hazard ratios for pairs with a Bonferroni-corrected *p*-value < 4.79 × 10^−4^ (=0.05/105). Statistical analyses were performed using the R program (version 3.6.3). We assigned a nominal *p*-value of < 0.05 as the significance level for most analyses, except the post hoc analyses.

## 3. Results

### 3.1. Characteristics of Study Population

In total, 4037 men and 4400 women were included in this study. Table 1 shows their baseline characteristics according to sex. The mean age was 51.6 ± 8.7 years in men, and 52.5 ± 9.0 years in women (*p* < 0.001). Mean BMI was significantly lower in men than in women. Mean waist circumference, SBP, DBP, fasting glucose, and fasting triglycerides were significantly higher in men than in women. Smoking and alcohol intake were also significantly more prevalent in men than in women.

### 3.2. Prevalence of MetSyn Components

**Figure 1:**
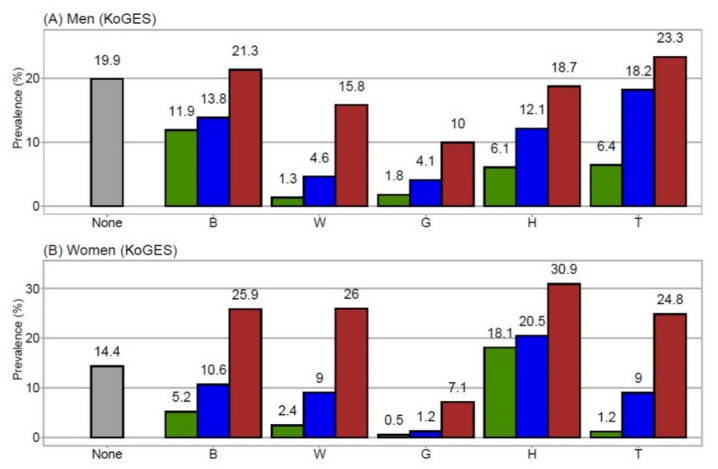
This is a figure. Schemes follow the same formatting. (**A**) prevalence in men, (**B**) prevalence in women. Grey bars indicate the prevalence of subjects with no MetSyn components (i.e., non-MetSyn patients). Green bars represent the prevalence of subjects with only one MetSyn component (B, W, G, H, or T). Blue bars represent the prevalence of subjects with a specific component plus one other MetSyn component. Brown bars represent the prevalence of subjects with a specific component plus two or more other MetSyn components (i.e., MetSyn patients). Numbers located above the bars indicate the numerical prevalence values. B, elevated blood pressure; G, elevated glucose; H, low HDL-cholesterol; KoGES, Korean Genome and Epidemiology Study Ansan and Ansung; MetSyn, metabolic syndrome; T, elevated triglycerides; W, increased waist circumference.

Figure 1A (men) and Figure 1B (women) display the prevalence of each MetSyn component alone, in combination with one other component (e.g., B+G, B+W, B+H, B+T), and in combination with two or more other components, fulfilling the definition of MetSyn. For each component, co-occurrence with at least one other component was more prevalent than the prevalence of the component alone.

**Figure 2:**
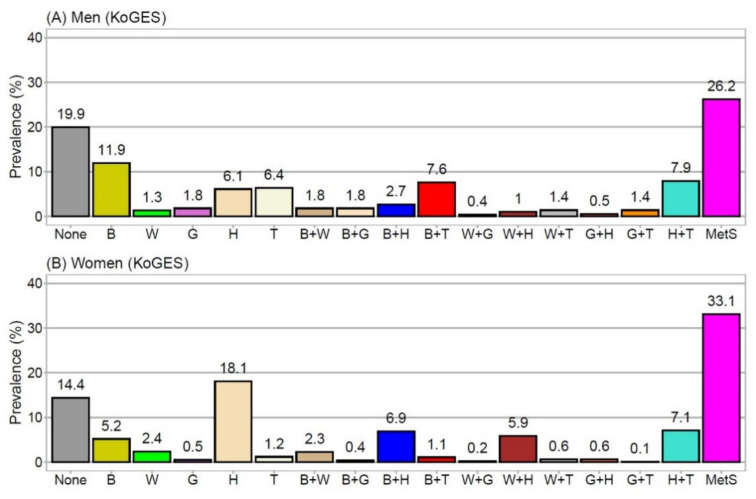
Prevalence of each MetSyn component, combinations of two MetSyn components, and MetSyn. (**A**) prevalence in men, (**B**) prevalence in women. Numbers located above the bars represent the numerical prevalence values. All groups are mutually exclusive; thus, the sum of prevalences was 100%. Colors for each component and combination are the same as those used in Figure 3 and Figure 4 to facilitate visual comparisons. B, elevated blood pressure; G, elevated glucose; H, low HDL-cholesterol; KoGES, Korean Genome and Epidemiology Study Ansan and Ansung; MetSyn, metabolic syndrome; T, elevated triglycerides; W, increased waist circumference.

Figure 2A (men) and Figure 2B (women) show the prevalence of each MetSyn component alone, the prevalence of two-component combinations, and the prevalence of MetSyn. In men, the prevalence of non-MetSyn, pre-MetSyn, and MetSyn was 19.9%, 54.0%, and 26.2%, respectively. In women, the prevalence of non-MetSyn, pre-MetSyn, and MetSyn was 14.4%, 52.6%, and 33.1%, respectively. The proportion of participants with no MetSyn components was significantly higher in men than in women.

The three most frequent single components were B, H, and T in men and B, W, and H in women. Among the 10 two-component combinations, the three most frequent combinations were H+T, B+T, and B+H in men (Figure 2A) and H+T, B+H, and W+H in women (Figure 2B).

### 3.3. Associations between MetSyn Components (Single or Two-Component Combinations) and New-Onset T2D or Hypertension

At baseline, 7433 participants did not have T2D and 5476 did not have hypertension. Over the 12-year follow-up period, a total of 1414 people developed new-onset T2D (men: 721; women: 693) and 1847 developed new-onset hypertension (men: 925; women: 922). Mean follow-up time to diagnosis of new-onset T2D was 8.78 years in men and 9.17 years in women. Mean follow-up time to diagnosis of new-onset hypertension was 8.01 years in men and 8.45 years in women.

**Figure 3:**
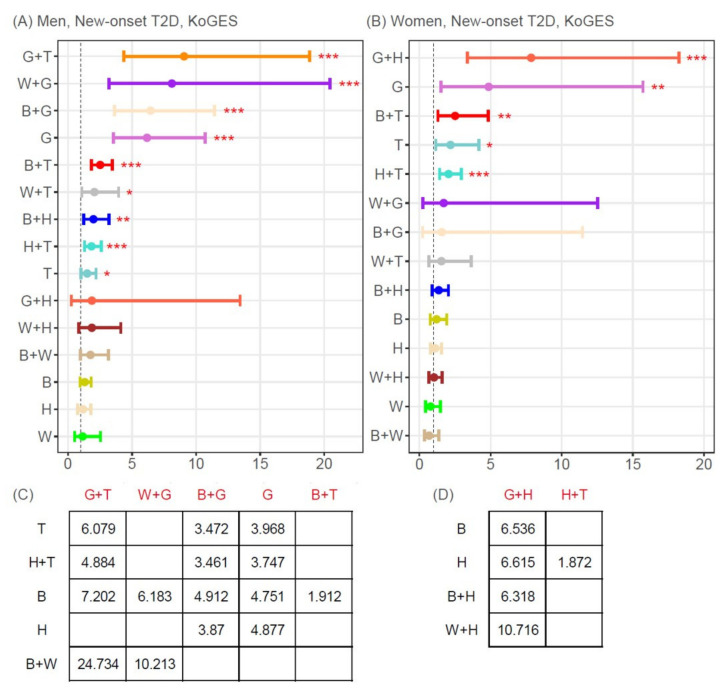
Adjusted hazard ratios and 95% confidence intervals for new-onset type2 diabetes. (**A**,**C**) men, (**B**,**D**) women. (**A**,**B**) Hazard ratios and 95% confidence intervals for the risk of new-onset type 2 diabetes of each single MetSyn component and two-component combination, compared with non-MetSyn, based on multivariate Cox regression analysis after adjusting for age, body mass index, smoking, and alcohol intake. Closed circles represent adjusted hazard ratios, and short vertical bars indicate the lower and upper limits of the 95% confidence interval for new-onset type 2 diabetes. (**C**,**D**) Adjusted hazard ratios for new-onset type 2 diabetes when setting the rows (black color; e.g., T, H+T, B]) and columns (red color; e.g., G+T, W+G, B+G) as reference and comparison groups, respectively. Of the 105 possible unique comparisons (based on a 15 × 15 matrix), adjusted hazard ratios are shown for only those comparisons with a Bonferroni-corrected *p*-value < 4.79 × 10^−4^ (=0.05/105). * *p* < 0.05, ** *p* < 0.01, *** *p* < 0.001. B, elevated blood pressure; G, elevated glucose; H, low HDL-cholesterol; KoGES, Korean Genome and Epidemiology Study Ansan and Ansung; MetSyn, metabolic syndrome; T, elevated triglycerides; T2D, type 2 diabetes; W, increased waist circumference.

**Figure 4:**
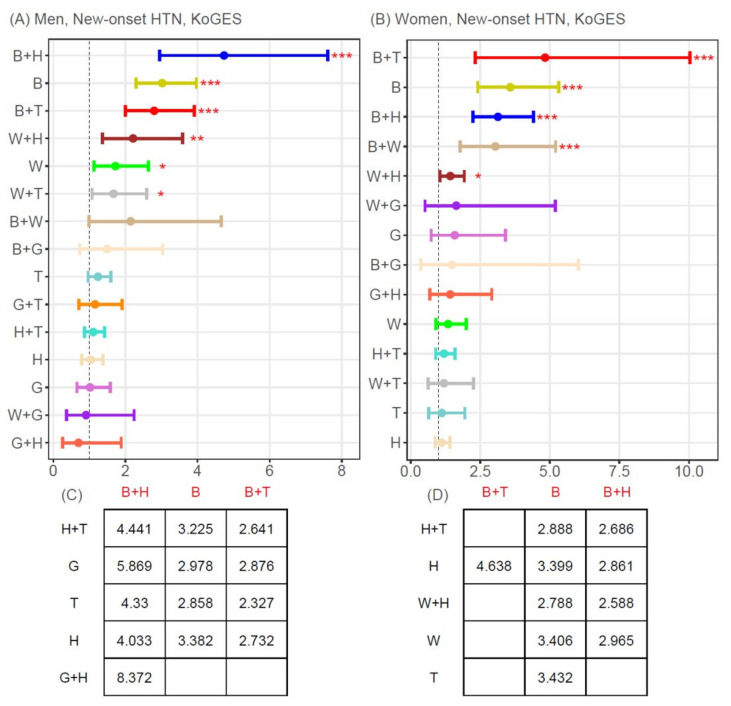
Adjusted hazard ratios and 95% confidence intervals for new-onset hypertension. (**A**,**C**) men, (**B**,**D**) women. (**A**,**B**) Hazard ratios and 95% confidence intervals for the risk of new-onset hypertension of each single MetSyn component and two-component combination, compared with non-MetSyn, based on multivariate Cox regression analysis after adjusting for age, body mass index, smoking, and alcohol intake. Closed circles represent adjusted hazard ratios, and short vertical bars indicate the lower and upper limits of the 95% confidence interval for new-onset hypertension. (**C**,**D**) Adjusted hazard ratios for new-onset hypertension when setting the rows (black color; e.g., B+H, G, T]) and columns (red color; e.g., B+H, B, B+T) as reference and comparison groups, respectively. Of the 105 possible unique comparisons (based on a 15 × 15 matrix), adjusted hazard ratios are shown for only those comparisons with a Bonferroni-corrected *p*-value < 4.79 × 10^−4^ (=0.05/105). * *p* < 0.05, ** *p* < 0.01, *** *p* < 0.001. B, elevated blood pressure; G, elevated glucose; H, low HDL-cholesterol; HTN, hypertension; KoGES, Korean Genome and Epidemiology Study Ansan and Ansung; MetSyn, metabolic syndrome; T, elevated triglycerides; W, increased waist circumference.

Figure 3 and Figure 4 display the results of the multivariate Cox proportional hazards regression analysis and post-hoc analysis for the incidence of new-onset T2D or hypertension for the 5 single MetSyn components and the 10 possible two-component combinations.

The following two-component combinations were associated with a significantly higher risk of new-onset T2D, compared to participants with no MetSyn components, after adjusting for age, BMI, smoking, and alcohol intake: G+T (HR 9.06 [95% CI 4.36–18.85]), W+G (HR 8.11 [95% CI 3.21–20.45]), B+G (HR 6.45 [95% CI 3.63–11.45]), B+T (HR 2.51 [95% CI 1.83–3.45]), W+T (HR 2.06 [95% CI 1.07–3.95]), B+H (HR 1.99 [95% CI 1.23–3.20]), and H+T (HR 1.83 [95% CI 1.30–2.59]) in men (Figure 3A) and G+H (HR 7.85 [95% CI 3.39–18.25]), B+T (HR 2.51 [95% CI 1.30–.83]), and H+T (HR 2.05 [95% CI 1.42–2.94]) in women (Figure 3B). As single components, G and T were significantly associated with new-onset T2D in both men and women.

The following two-component combinations were associated with a significantly higher risk of new-onset hypertension, after adjusting for age, BMI, smoking, and alcohol intake: B+H (HR 4.74 [95% CI 2.95–7.61]), B+T (HR 2.80 [95% CI 2.00–3.91]), W+H (HR 2.21 [95% CI 1.37–3.59]), W+T (HR 1.67 [95% CI 1.08–2.59]) in men (Figure 4A) and B+T (HR 4.83 [95% CI 2.32–10.04]), B+H (HR 3.14 [95% CI 2.23–4.41]), B+W (HR 3.04 [95% CI 1.78–5.21]), and W+H (HR 3.04 [95% CI 1.78–5.21]) in women (Figure 4B). As single components, B and W were significantly related with new-onset hypertension in men, and only B was significantly related with new-onset hypertension in women.

## 4. Discussion

The present study showed that the prevalence of one or two MetSyn components (i.e., pre-MetSyn) was higher than the prevalence of no MetSyn components or MetSyn in both men and women. We also found that single MetSyn components or combinations of two MetSyn components were differently associated with increased risks of developing new-onset T2D or hypertension. In addition, MetSyn components (alone or as two-component combinations) associated with new-onset T2D or hypertension differed between men and women.

Several previous studies have reported different diagnostic criteria for pre-MetSyn, the precursor of MetSyn [14,15,16]. In their study of 160 adolescent women in China, Yin et al. [14] defined pre-MetSyn as the presence of at least two components of MetSyn that did not reach the actual criteria threshold for MetSyn. Vidigal et al. [16] used the same definition for pre-MetSyn in the LATIN America METabolic Syndrome (LATINMETS) multicenter study of 226 adults aged 20–59 years. However, these studies were limited by their small size and lack of sex-specific assessments. Another study [15], consisting of 607 subjects aged 21 years and older, defined pre-MetSyn as the presence of one or two components of MetSyn. Our results showed that various single MetSyn components and two-component combinations were significantly associated with the development of incident T2D and hypertension, when compared with no MetSyn components. Therefore, we suggest that pre-MetSyn be defined as the presence of one or two MetSyn components. Moreover, only 19.9% of men and 14.4% of women had no MetSyn components at baseline, and more than 50% of all participants had one or two MetSyn components, which we defined as pre-MetSyn. These results, therefore, suggest that pre-MetSyn is common and warrants more attention from public health professionals.

Single MetSyn components and two-component combinations associated with an increased risk of new-onset T2D or hypertension (compared with the absence of all MetSyn components) differed according to disease and, to a lesser extent, sex. The prevalence of single MetSyn components and two-component combinations also differed by sex. Furthermore, in pairwise comparisons of the 15 single MetSyn components and two-component combinations, with respect to the risk of new-onset MetSyn, most comparisons showed no significant differences between components (post-hoc analyses; Supplementary Figure S2). These results suggest that it is difficult to emphasize specific components or combinations as the most important risk factor for chronic disease, which is consistent with current medical opinions that there should be no obligatory component for diagnosing MetSyn [5].

Although heterogeneous relationships were observed according to disease and sex, a number of similarities between sexes were observed. For new-onset T2D, G and T as single components were significant risk factors in both men and women. Of the two-component combinations, seven were significant risk factors in men (G+T, W+G, B+G, B+T, W+T, B+H, and H+T) and three were significant risk factors in women (G+H, B+T, and H+T). Not all four possible combinations containing the G component were significantly associated with an increased risk of T2D, which is inconsistent with the results of previous studies [7,11,19,20]. Among the combinations with no G component, B+T, W+T, B+H, and H+T in men and B+T and H+T in women were significantly associated with new-onset T2D. Of note, most of these combinations included the T component. This is consistent with the results of a stratified analysis of associations between all combinations of MetSyn components and T2D in Korean adults, which found that H+T and B+T were the two-component combinations excluding G with the strongest associations with T2D [11]. Thus, elevated serum triglycerides appear to be an important risk factor for new-onset T2D. One possible explanation for this observation is the relationship between increased serum triglycerides and insulin resistance [21,22].

For new-onset hypertension, B and W were single risk factors in men, whereas B was the only significant risk factor in women. Of the two-component combinations, four were significant risk factors in men (B+H, B+T, W+H, and W+T) and four were significant risk factors in women (B+H, B+T, W+H, and B+W). Among the combinations not including B, W+H and W+T in men and W+H in women were significantly associated with new-onset hypertension. In previous studies examining individual MetSyn components and the risk of hypertension [23,24], central obesity was reported as the second most important component after hypertension, which may be explained by several mechanisms, including sympathetic nervous system over-activation, stimulation of the renin–angiotensin–aldosterone system, and alterations in adipose-derived cytokines [25]. Although it is difficult to unify pathophysiological mechanisms resulting in T2D and hypertension, abdominal adiposity and insulin resistance appear to be central to MetSyn and its individual components.

The current study had some limitations. First, it did not consider the effects of sequential changes in MetSyn components and other lifestyle factors. Second, the study population may not represent the general Korean population, since the participants were specific community dwellers. Differences in race, age, sex, and definition of MetSyn components could also lead to inconsistent findings. Lastly, although we examined associations after adjusting for potential confounding factors, we could not completely exclude the effects of other unexamined confounding variables, such as a family history of diabetes or hypertension and level of physical activity.

Our study also had several strengths. It had a prospective cohort study design, and the follow-up period was relatively long (12 years). Furthermore, we performed analyses according to sex, unlike most other previous studies. Our large population-based sample was also a major strength and allowed us to suggest an appropriate definition for pre-MetSyn.

## 5. Conclusions

In conclusion, additional studies including various populations and ethnicities are required to more fully define pre-MetSyn, which is an important precursor for the development of chronic diseases. The pre-MetSyn state could be the best time to initiate effective strategies to prevent the development of T2D and hypertension.

## Figures and Tables

**Table 1:** Baseline characteristics of the study population.

	Men	Women	*p*-Value
Number	4037	4400	
Age (y)	51.6 ± 8.7	52.5 ± 9.0	<0.001
Body mass index (kg/m^2^)	24.2 ± 2.9	24.9 ± 3.3	<0.001
Waist circumference (cm)	83.6 ± 7.7	81.7 ± 9.6	<0.001
Systolic blood pressure (mmHg)	122.4 ± 17.0	121.3 ± 19.8	0.004
Diastolic blood pressure (mmHg)	82.2 ± 11.1	79.1 ± 12.0	<0.001
Fasting glucose (mg/dL)	89.8 ± 22.8	84.7 ± 18.1	<0.001
Total cholesterol (mg/dL)	191.8 ± 35.6	191.3 ± 35	0.568
Triglycerides (mg/dL)	176.6 ± 118.1	146.2 ± 83.7	<0.001
HDL-cholesterol (mg/dL)	43.6 ± 10.0	45.8 ± 10.0	<0.001
Hypertension, *n* (%)	529 (13.1)	721 (16.4)	<0.001
Diabetes, *n* (%)	217 (5.4)	138 (3.1)	<0.001
Smoking, *n* (%)	1780 (44.1)	105 (2.4)	<0.001
Alcohol intake, *n* (%)	18.6 ± 28.4	1.4 ± 6.2	<0.001

Data are mean ± standard deviation except where indicated otherwise. HDL, high-density lipoprotein.

## Data Availability

The data can be accessed on the http://www.nih.go.kr/contents.es?mid=a40504060100, accessed on 29 May 2020.

## Supplementary Materials

The following are available online at https://www.mdpi.com/article/10.3390/jpm11080700/s1, Supplementary Figure S1 Flow chart of the study population selection process. Supplementary Figure S2 Adjusted hazard ratios for new-onset metabolic syndrome. (A) men, (B) women. Hazard ratios and 95% confidence intervals for new onset metabolic syndrome in each single MetSyn component and two-component combination compared to non-MetSyn were calculated using the multivariate cox regression analysis after adjusting for age, BMI, alcohol, and smoking. Point, left and right vertical bars indicate adjusted hazard ratios and lower and upper 95% confidence intervals for new onset metabolic syndrome, respectively. The matrix in the lower part of the figure indicates the adjusted hazard ratio for new onset metabolic syndrome, setting row (black color [e.g., T, H+T, and B]) and column (red color) groups as the reference and comparison groups, respectively. Among a 15 (5C1+5C2) by 15 matrix, we illustrate the results (adjusted hazard ratios) with significant power as Bonferroni-corrected *p*-value < 0.05 [uncorrected *p*-value = 4.79 × 10^−4^ (=0.05/105)]. ** *p* < 0.01, *** *p* < 0.001. B, elevated blood pressure; BMI, body mass index; H, low HDL-cholesterol; MetSyn, metabolic syndrome; T, elevated triglycerides.
