# Supplementary figures and images for: Pre-Metabolic Syndrome and Incidence of Type 2 Diabetes and Hypertension: From the Korean Genome and Epidemiology Study
Source: J Pers Med. 2021 Jul 22;11(8):700. doi: 10.3390/jpm11080700 (PMC8398139; doi:10.3390/jpm11080700)

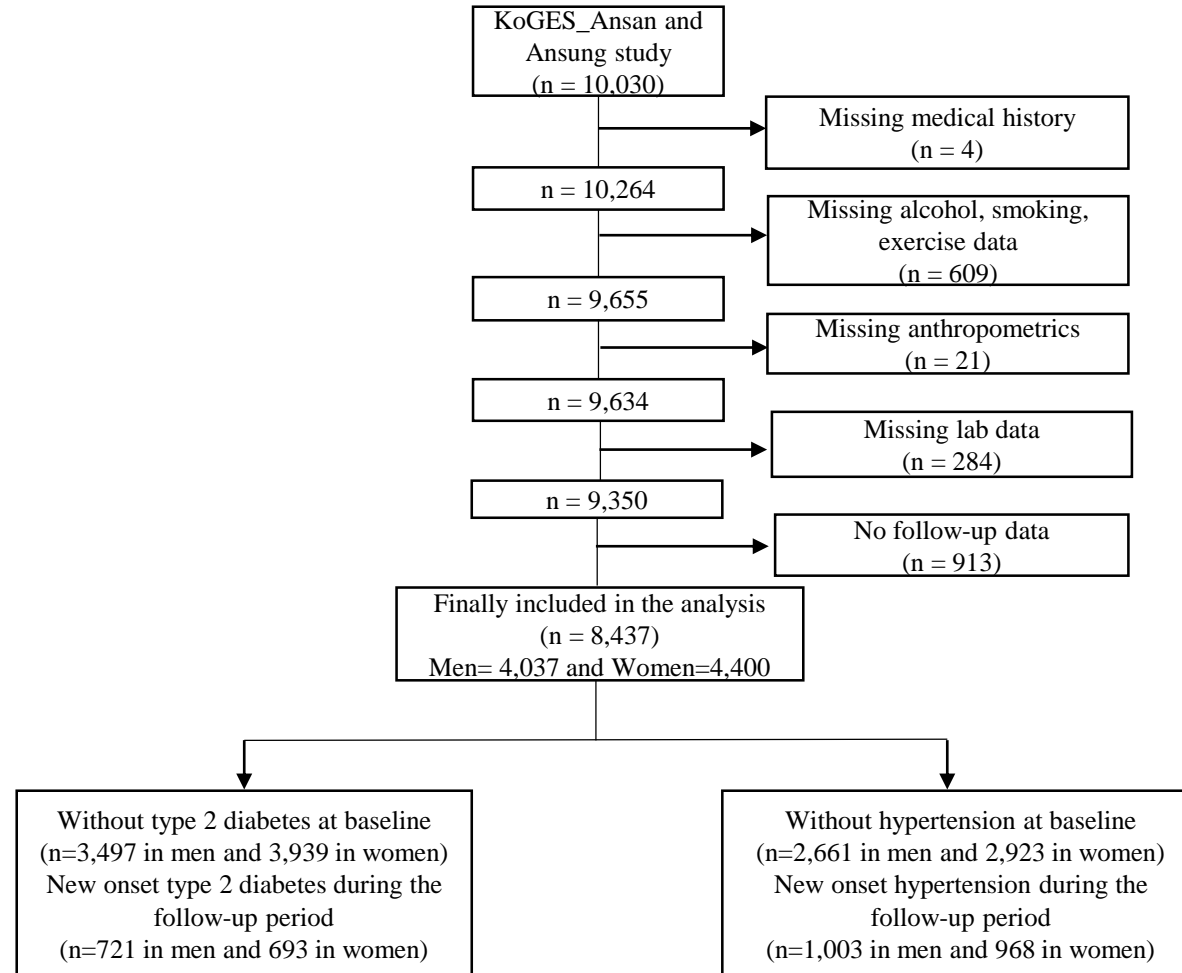

Supplement: Supplementary file 1 [file jpm-11-00700-s001.zip › Supplementary Figure S1.pdf]

(A) Men, New-onset MetSyn, KoGES

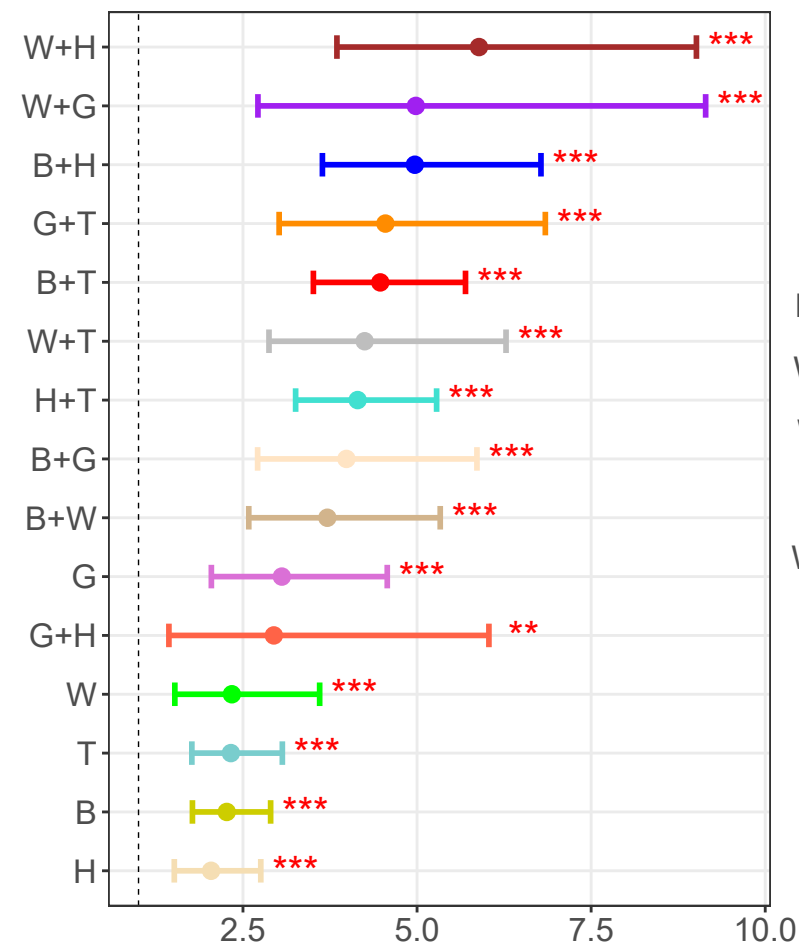

(B) Women, New-onset MetSyn, KoGES

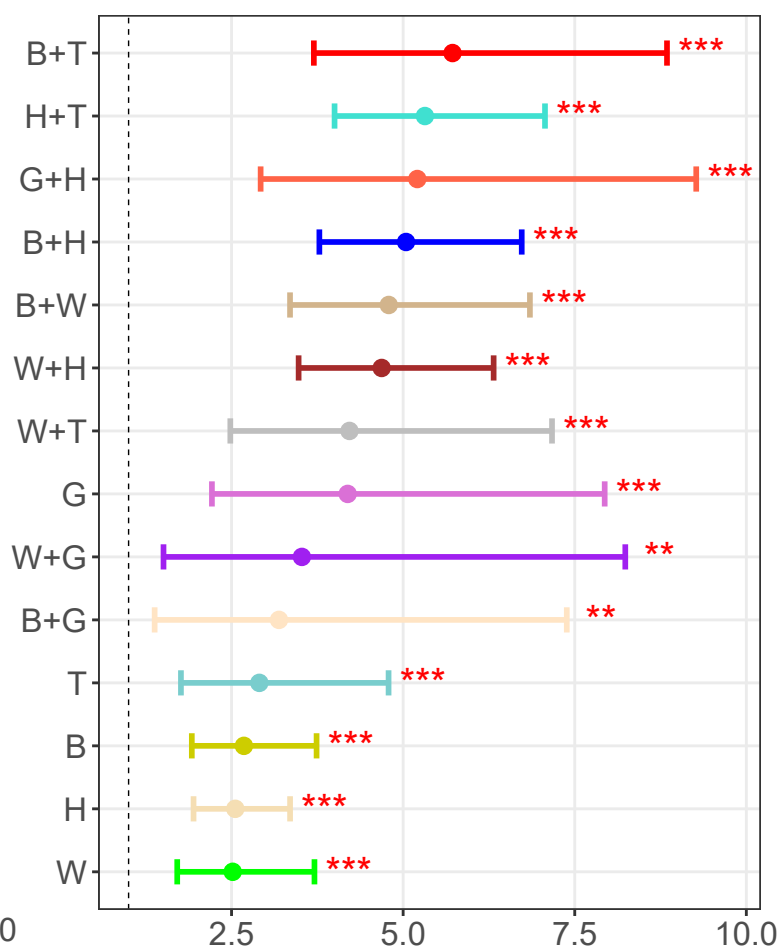

(C) W+H B+H G+T B+T H+T

|   |       |       |      |       |       |
|---|-------|-------|------|-------|-------|
| B |       | 2.112 | 2.11 | 1.983 | 1.753 |
| T | 3.452 | 2.491 |      | 2.072 | 1.894 |
| H |       | 2.22  |      | 2.383 | 1.995 |

(D) B+T H+T B+H W+H

|   |       |       |      |       |
|---|-------|-------|------|-------|
| H | 2.271 | 2.043 | 1.95 | 1.606 |
| B | 2.214 | 1.956 | 1.8  | 1.829 |

Supplement: Supplementary file 1 [file jpm-11-00700-s001.zip › Supplementray Figure S2.pdf]
